# A vigilância genômica do SARS-CoV-2 no Brasil na resposta à pandemia da COVID-19

**DOI:** 10.26633/RPSP.2021.75

**Published:** 2021-05-26

**Authors:** Janaína Sallas, Guilherme Almeida Elidio, Daniela Buosi Rohlfs, Arnaldo Correia de Medeiros, Dirce Bellezi Guilhem

**Affiliations:** Faculdade de Ciências da Saúde, Programa de Pós-Graduação de Ciências da Saúde, Universidade de Brasília Brasília (DF) Brasil Faculdade de Ciências da Saúde, Programa de Pós-Graduação de Ciências da Saúde, Universidade de Brasília, Brasília (DF), Brasil; Ministério da Saúde, Secretaria de Vigilância em Saúde Brasília, (DF) Brasil Ministério da Saúde, Secretaria de Vigilância em Saúde, Brasília, (DF), Brasil.

Ao Editor,

Diariamente, milhares de variantes do SARS-CoV-2 surgem no mundo (1), evidenciando o desafio de reforçar a capacidade de detecção oportuna com ampliação e fortalecimento da vigilância epidemiológica, vigilância laboratorial e comunicação rápida, com desenvolvimento de pesquisas em saúde que possam apoiar as medidas de prevenção e controle da pandemia da COVID-19. Ainda que não se conheçam todas as implicações das novas variantes da COVID-19 para o controle da doença, algumas mutações ou combinações podem fornecer ao vírus uma vantagem seletiva, que contribui para aumentar a transmissibilidade ou a capacidade de evadir a resposta imune do hospedeiro.

No Brasil, até 10 de abril de 2021, foram registrados e confirmados 13 445 006 casos e 361 334 óbitos por COVID-19, com incidência acumulada de 6 397,9 por 100 mil habitantes e mortalidade de 157,2 por 100 mil habitantes. De acordo com a Organização Mundial da Saúde (OMS), o Brasil é o segundo país com maior número de casos e óbitos por COVID-19 no mundo (2). Entretanto, além da alta incidência e mortalidade pela doença encontradas no Brasil, o Sistema Único de Saúde (SUS) está sendo desafiado pela circulação de variantes de atenção do SARS-CoV-2 nos estados.

No cenário mundial, existem três variantes de atenção para SARS-CoV-2 (3): do Reino Unido (B.1.1.7), com circulação em 111 países; da África do Sul (B.1.351), com circulação em 70 países; e do Brasil (P.1.), com circulação em 36 países. No Brasil, no período compreendido pelas semanas epidemiológicas 1 até 14 de 2021 (ou seja, de 3 de janeiro de 2021 até 10 de abril de 2021), a vigilância genômica identificou essas três variantes de atenção. Quanto à P.1, variante de maior circulação no Brasil, detectada pelas autoridades em 9 de janeiro de 2021, 1 690 amostras foram registradas em 24 unidades federadas nas cinco regiões do país, sendo 44,6% originárias da região Norte. Com relação à B.1.1.7, foi detectada pelas autoridades brasileiras em 2 de janeiro de 2021 e, até o momento, foram identificadas 63 amostras distribuídas em 10 unidades federadas e quatro regiões (Nordeste, Sudeste, Sul e Centro Oeste), porém com concentração no Sudeste (63,5%). Quanto à B.1.351, apenas um caso foi detectado no país, em 7 de abril de 2021, em São Paulo, região Sudeste.

Os estudos sobre a variante de atenção P.1. ainda são muito limitados, havendo poucas evidências disponíveis para determinar uma mudança na transmissibilidade ou gravidade da doença (4). No entanto, é plausível o aumento da transmissibilidade, tendo em vista a presença de alteração de aminoácidos N501Y nas três variantes de atenção (5, 6). Com relação à B.1.1.7, pesquisas do Reino Unido estimam ser até 71% mais transmissível do que a forma anteriormente circulante do vírus, com possibilidade de carga viral mais alta (6). No rastreamento de contatos, a taxa de ataque secundário pode ser maior, de 15,1% *versus* 9,8% em outras variantes (6). Estudos afirmam que a variante de atenção B.1.351 pode apresentar maior potencial de transmissibilidade, cerca de 50%, em comparação com as variantes pré-existentes, além de ser amplamente resistente a anticorpos neutralizantes, podendo gerar risco significativo de reinfecção (6).

Nesse cenário, muitas perguntas precisam ser respondidas de forma oportuna, como: existem diferenças na doença provocada pelas variantes de atenção? Como as variantes podem afetar a assistência, as práticas terapêuticas, a resposta às vacinas e os testes disponíveis? Em busca de respostas, desde 8 de fevereiro de 2021 o Ministério da Saúde do Brasil está implementando um plano nacional de fortalecimento e ampliação da vigilância genômica para SARS-CoV-2. Entre os objetivos propostos estão: identificar estratégias de vigilância genômica; orientar quanto aos critérios para sequenciamento de amostras; padronizar o envio dos resultados das pesquisas genômicas; reforçar a comunicação imediata e oportuna; e analisar os resultados das pesquisas genômicas em parceria com a vigilância epidemiológica. Em um prazo de 14 dias, a rede de vigilância genômica passou de 10 amostras mensais por estado para três mil amostras a cada 4 semanas epidemiológicas. Acreditamos que o fortalecimento da rede mundial de vigilância genômica seja um dos caminhos para subsidiar as medidas de controle da pandemia da COVID-19 em todo o mundo, tornando possível o enfrentamento do desafio das variações do SARS-CoV-2 no SUS e no Brasil.!

## Declaração.

As opiniões expressas no manuscrito são de responsabilidade exclusiva dos autores e não refletem necessariamente a opinião ou política da RPSP/PAJPH ou da Organização Pan-Americana da Saúde (OPAS).
